# Added value of histogram analysis of intravoxel incoherent motion and diffusion kurtosis imaging for the evaluation of complete response to neoadjuvant therapy in locally advanced rectal cancer

**DOI:** 10.1007/s00330-024-11081-z

**Published:** 2024-09-19

**Authors:** Lan Zhang, Ziwei Jin, Fan Yang, Yiwan Guo, Yuan Liu, Manman Chen, Si Xu, Zhenyu Lin, Peng Sun, Ming Yang, Peng Zhang, Kaixiong Tao, Tao Zhang, Xin Li, Chuansheng Zheng

**Affiliations:** 1 Department of Radiology, Union Hospital, Tongji Medical College, Huazhong University of Science and Technology, Wuhan, Hubei 430022 China; 2 Hubei Province Key Laboratory of Molecular Imaging, Wuhan, Hubei 430022 China; 3 Cancer Center, Union Hospital, Tongji Medical College, Huazhong University of Science and Technology, Wuhan, Hubei 430022 China; 4 Institute of Radiation Oncology, Union Hospital, Tongji Medical College, Huazhong University of Science and Technology, Wuhan, Hubei 430022 China; 5 Hubei Key Laboratory of Precision Radiation Oncology, Wuhan, Hubei 430022 China; 6 Clinical and Technical Support, Philips Healthcare, Beijing, 100600 China; 7 Department of Pathology, Union Hospital, Tongji Medical College, Huazhong University of Science and Technology, Wuhan, Hubei 430022 China; 8 Department of Gastrointestinal Surgery, Union Hospital, Tongji Medical College, Huazhong University of Science and Technology, Wuhan, Hubei 430022 China

**Keywords:** Diffusion magnetic resonance imaging, Rectal cancer, Neoadjuvant therapy, Complete response

## Abstract

**Objective:**

To evaluate how intravoxel incoherent motion (IVIM) and diffusion kurtosis imaging (DKI) histogram analysis contribute to assessing complete response (CR) to neoadjuvant therapy (NAT) in locally advanced rectal cancer (LARC).

**Material and methods:**

In this prospective study, participants with LARC, who underwent NAT and subsequent surgery, with adequate MR image quality, were enrolled from November 2021 to March 2023. Conventional MRI (T2WI and DWI), IVIM, and DKI were performed before NAT (pre-NAT) and within two weeks before surgery (post-NAT). Image evaluation was independently performed by two experienced radiologists. Pathological complete response (pCR) was used as the reference standard. An IVIM–DKI-added model (a combination of IVIM and DKI histogram parameters with T2WI and DWI) was constructed. Receiver operating characteristic (ROC) curves were generated to evaluate the diagnostic performance of conventional MRI and the IVIM–DKI-added model.

**Results:**

A total of 59 participants (median age: 58.00 years [IQR: 52.00, 62.00]; 38 [64%] men) were evaluated, including 21 pCR and 38 non-pCR cases. The histogram parameters of DKI, including skewness of kurtosis post-NAT (post-*K*_Skewness_) and root mean squared of change ratio of diffusivity (Δ%*D*_DKI-root mean squared_), were entered into the IVIM–DKI-added model. The area under the ROC curve (AUC) of the IVIM–DKI-added model for assessing CR to NAT was significantly higher than that of conventional MRI (0.855 [95% CI: 0.749–0.960] vs 0.685 [95% CI: 0.565–0.806], *p* < 0.001).

**Conclusion:**

IVIM and DKI provide added value in the evaluation of CR to NAT in LARC.

**Key Points:**

***Question*** *The current conventional imaging evaluation system lacks adequacy for assessing CR to NAT in LARC*.

***Findings*** *Significantly improved diagnostic performance was observed with the histogram analysis of IVIM and DKI in conjunction with conventional MRI*.

***Clinical relevance*** *IVIM and DKI provide significant value in evaluating CR to NAT in LARC, which bears significant implications for reducing surgical complications and facilitating organ preservation*.

## Introduction

Neoadjuvant therapy (NAT) along with total mesorectal excision has become the established treatment for locally advanced rectal cancer (LARC) [1]. Approximately 10–25% of patients achieve pathological complete response (pCR) following NAT [2, 3]. With a local regrowth rate of 30% at 5 years [4]. A watch-and-wait strategy is increasingly employed for patients showing favorable responses to NAT, particularly for those desiring sphincter preservation [5]. Accurate assessment of tumor response is crucial for definitive treatment decisions, as the evaluation of complete response (CR) post-NAT plays a pivotal role in clinical management.

Standard methods for assessing CR include digital rectal examination (DRE), endoscopy, and MRI. However, DRE and endoscopy provide limited visualization, often failing to detect residual tumors within or around the rectal wall [6, 7]. Conventional MRI, incorporating high-spatial-resolution T2-weighted imaging (T2WI) [8] and diffusion-weighted imaging (DWI) [9–11], is pivotal for CR assessment by revealing information regarding residual tumors in the rectal wall vicinity. Nonetheless, the diagnostic efficacy of conventional MRI falls short of clinical demand, with meta-analytic summary sensitivity of 0.62 (95% confidence interval (CI): 0.43–0.77) and summary specificity of 0.89 (95% CI: 0.80–0.94) [12]. Conventional MRI’s limitations stem from T2WI’s inability to accurately differentiate residual tumor from post-therapeutic fibrosis and edema [13], and DWI’s reliance on the assumption of unrestricted free diffusion, which does not accurately reflect water motion in biological tissues exhibiting non-Gaussian diffusion. Additionally, the apparent diffusion coefficient (ADC) has demonstrated inconsistent diagnostic performances in prior studies [14]. Hence, there is an urgent need for imaging evaluation methods offering higher precision.

As advanced diffusion models [15, 16], diffusion kurtosis imaging (DKI) provides insights into tissue structural connectivity, while intravoxel incoherent motion (IVIM) offers information on microvascular perfusion and diffusion of water molecules within living tissues. These techniques offer valuable quantitative data on tumor characteristics and have found wide application in assessing various cancers such as breast cancer [17, 18], cerebral glioma [19, 20], and prostate cancer [21, 22]. In the context of rectal cancer, IVIM and DKI have been utilized for staging, predicting lymph node metastasis, assessing prognosis, and evaluating therapy response to NAT [23–26].

Recognizing that both IVIM and DKI may contribute valuable information in therapy response evaluation, our study employed a “one-stop” IVIM–DKI scanning protocol to acquire IVIM and DKI data in a single scan. This approach reduces data redundancy, shortens scan time, and mitigates motion-related artifacts. However, the diagnostic performance of this “one-stop” IVIM–DKI scanning protocol in the context of multiparametric MRI analysis for evaluating CR to NAT remains unknown. Therefore, our aim is to assess the added value of IVIM–DKI parameters combined with first-order texture feature analysis in the evaluation of CR to NAT in LARC.

## Materials and methods

### Participants

This prospective study received approval from the institutional review board of Union Hospital affiliated with Tongji Medical College of Huazhong University of Science and Technology. All participants provided informed consent. Between November 2021 and March 2023, consecutive patients diagnosed with rectal adenocarcinoma were initially enrolled. Patients meeting the following criteria were included: (1) aged 18 to 75 years; (2) underwent routine MRI, IVIM–DKI examinations at baseline, and before surgery; (3) clinically and histologically confirmed non-mucinous LARC (T3-4N0M0 or T1-4N + M0); and (4) scheduled for NAT followed by TME. Exclusion criteria were as follows: (1) history of or concurrent other malignancy; (2) time interval between NAT end and surgical procedure > 10 weeks; (3) time interval between post-NAT MRI and surgery > 2 weeks; (4) no TME; and (5) inadequate MRI quality.

### MRI acquisition

All participants underwent imaging using a 3-T clinical scanner (Ingenia 3.0-T, Philips Healthcare) with a 32-channel phased-array body coil in the supine position twice, respectively, before NAT (pre-NAT) and within two weeks before surgery (post-NAT). To minimize bowel motion, participants received 20 mg of raceanisodamine hydrochloride injection intramuscularly 30 min prior to the MR examination. The IVIM–DKI sequence was acquired in the oblique axial plane using a spectral presaturation with inversion recovery (SPIR) fat-suppressed single-shot echo-planar imaging (EPI) pulse sequence. The imaging protocol (Table 1) comprised T2WI, DWI, and IVIM–DKI.

**Table 1:** Details of sequence parameters

Sequence	T2WI	DWI	IVIM–DKI
Imaging technique	TSE	EPI	EPI
Repetition time (ms)	4134	1983	3411
Echo time (ms)	100	62	92
Flip angle (degrees)	90	90	90
Field of view (mm^2^)	200 × 200	370 × 303	300 × 262
Matrix (frequency × phase)	288 × 231	132 × 108	152 × 105
Spatial resolution (mm^2^)	0.7 × 0.8	2.8 × 2.78	2.0 × 2.5
Slice thickness (mm)	3	6	5
Slice gap (mm)	0.3	1	1
Bandwidth (kHz)	217.6	34.9	26.7
Packages	2	2	1
No. of slices	36	32	20
ETL/TFE factor	22	45	53
Fat suppression technique	N/A	SPIR	SPIR
Respiratory control	Free-breathing	Free-breathing	Free-breathing
Imaging time (min:s)	2:25	1:00	5:24

*T2WI* T2-weighted MRI, *DWI* diffusion-weighted MRI, *IVIM* intravoxel incoherent motion, *DKI* diffusion kurtosis imaging, *TSE* turbo spin echo, *EPI* echo planar imaging, *TFE* turbo field echo, *ETL* echo train length, *SPIR* spectral presaturation with inversion recovery

### Imaging post-process

Initially, we conducted an image quality assessment to ensure the stability of parameter measurements. The quality of T2WI, DWI, and IVIM–DKI images was evaluated by two experienced radiologists, blinded to the clinical and histopathologic results of the patients, using predefined criteria (Tables E2–4). Images scoring ≥ 3 were considered suitable for further analysis. Subsequently, the acquired pre- and post-NAT images were transferred in DICOM format to IntelliSpace Portal, version 10 (Philips Healthcare). DICOM images from the IVIM–DKI acquisition underwent post-processing using a vendor-provided advanced diffusion analysis (ADA) tool. We selected appropriate *b* values and models in ADA and derived quantitative images of IVIM and DKI. IVIM utilized a total of nine *b* values (0 s/mm^2^, 20 s/mm^2^, 40 s/mm^2^, 80 s/mm^2^, 160 s/mm^2^, 400 s/mm^2^, 600 s/mm^2^, 800 s/mm^2^, and 1000 s/mm^2^), with corresponding numbers of averages of 1, 1, 1, 1, 2, 2, 3, 3, and 4. Meanwhile, DKI employed a total of five *b* values (0 s/mm^2^, 800 s/mm^2^, 1000 s/mm^2^, 1500 s/mm^2^, and 2000 s/mm^2^), with corresponding numbers of averages of 1, 3, 4, 6, and 8.

The diffusivity values of DKI encompassed diffusivity (*D*) and kurtosis (*K*), and the relationship between DKI signal intensity and b factors can be expressed as:

$$S\left(b\right)={S}_{0}\cdot \exp (-{bD}+{b}^{2}{D}^{2}K/6)$$

where *S*(*b*) is the signal intensity at a specific *b* value, *S*_*0*_ is the signal intensity at *b* = 0 s/mm^2^, *D* is corrected ADC without Gaussian bias, and *K* is a unitless parameter that represents the deviation of water motion from Gaussian diffusion.

The diffusivity values of IVIM include slow diffusion coefficient (*D*), fast diffusion coefficient (D^*^), and perfusion-related diffusion fraction (*f*). The IVIM model and its parameters were fitted according to the following bi-exponential equation:

$$S(b)/{S}_{0}=f\cdot \exp (-{bD}* )+(1-f)\cdot \exp (-{bD})$$

where *S*(*b*) is the signal intensity at a specific b value, *S*_*0*_ is the signal intensity at *b* = 0 s/mm^2^, *f* represents perfusion fraction. D^*^ is the perfusion-related diffusion coefficient, and *D* represents the diffusion of the non-perfusing fraction.

### Tumor segmentation

Subsequently, the IVIM–DKI DICOM images generated by ADA postprocessing for each patient pre- and post-NAT were processed using 3D Slicer version 5.4.0 (www.slicer.org), a freely available open-source software. The IVIM–DKI images were overlaid onto the corresponding T2WI time scan. Radiologists combined structural information from T2WI with functional information from IVIM–DKI to delineate regions of interest (ROIs). These ROIs encompassed the rectal wall while excluding the lumen, submucosal edema, and necrosis, targeting the area of the largest suspicious residual tumor based on corresponding T2WI. In cases where no suspicious residual tumor was apparent on post-NAT T2WI, ROIs were drawn at the site of the largest initial lesion, referring to pre-NAT T2WI.

### Feature extraction

Histogram parameters of diffusivity of DKI (*D*_DKI_), *K*, true diffusion coefficient (*D*_IVIM_), D^*^, and *f* maps were extracted using the radiomics plugin in 3D Slicer. Each map yielded eighteen histogram parameters (see Supplemental Material for details of each parameter), comprising 10th percentile, 90th percentile, energy, entropy, interquartile range, kurtosis, maximum, mean absolute deviation, mean, median, minimum, range, robust mean absolute deviation, root mean squared, skewness, total energy, uniformity, and variance. A total of 270 features were obtained for each patient, encompassing 18 histogram parameters from DKI and IVIM parameters pre-NAT, post-NAT, and the change ratio. Extreme values of histogram parameters, such as minimum and maximum values, were excluded to mitigate the impact of image noise or artifacts. The representation of quantitative parameters *D*_DKI_, *K*, *D*_IVIM_, D^*^, and *f* maps pre- and post-NAT for each patient can be expressed as pre-*X* and post-*X*, *X* represents a certain quantitative parameter. The change ratio can be represented as Δ*X*%, calculated as Δ*X*% = (Post-*X* − pre-*X*)/pre-*X* × 100, with the subscript indicating the specific parameter among the 18 histogram parameters.

### Statistical analysis

Statistical analysis and graphing were performed using SPSS 25.0 (IBM), MedCalc statistical software version 22.0 (https://www.medcalc.org), and R version 4.3.2 (R Foundation for Statistical Computing). The Kolmogorov–Smirnov test was initially employed to assess the normality of continuous variables. Enumeration data differences were analyzed using the chi-squared or Fisher’s exact probability test, while continuous variables were compared using the Mann–Whitney *U*-test or the independent samples *t*-test.

For feature selection of histogram parameters, variables were chosen based on two main criteria: (1) significant between-group differences (*p* < 0.05 after multiple comparison correction) (Table E1); (2) low Spearman correlation coefficient between variables (< 0.4) (Fig. E3). An IVIM–DKI-added model (comprising IVIM–DKI histogram parameters, T2WI, and DWI) was constructed using predicted probabilities obtained from multivariate logistic regression analysis.

In MedCalc 22.0, receiver operating characteristic (ROC) curves were employed to assess the diagnostic performance of conventional MRI and the IVIM–DKI-added model. The optimal cutoff value was determined using the Youden index to calculate sensitivity and specificity for differential diagnosis. The Delong method was utilized to compare the area under the curve (AUC) of conventional MRI and the IVIM–DKI-added model.

For evaluating image quality and conventional MRI’s ability to detect CR to NAT based on combined T2WI and DWI, interobserver consistency was assessed using the kappa analysis. Intraclass correlation coefficients (ICCs) were calculated to evaluate interobserver agreement for IVIM–DKI parameters: 0.000–0.399, poor agreement; 0.400–0.599, fair agreement; 0.600–0.799, good agreement; 0.800–1.000, excellent agreement. All statistical tests were two-sided, with the test level set at α = 0.05.

### Histopathological examination

In our study, histopathological examination of surgical specimens served as the reference standard. All TME specimens underwent analysis following previously established protocols [27, 28]. Two senior gastrointestinal pathologists independently assessed all sections. The tumor regression grade (TRG) was determined according to criteria proposed by the American Joint Committee on Cancer, categorized as follows: TRG 0, no residual cancer cells; TRG 1, single or small groups of cancer cells; TRG 2, residual cancer with desmoplastic response; TRG 3, minimal evidence of tumor response. The extent of residual tumor was classified per the 8th edition of the International Union Against Cancer TNM staging system. pCR was defined as the absence of any viable cancer cells in the resected primary tumor specimen and all collected regional lymph nodes (ypT0N0). As this preliminary study focused solely on the treatment response of the primary tumor excluding regional lymph nodes, pCR is referred to as TRG 0.

## Results

### Patient demographic and clinical characteristics

**Fig. 1:**
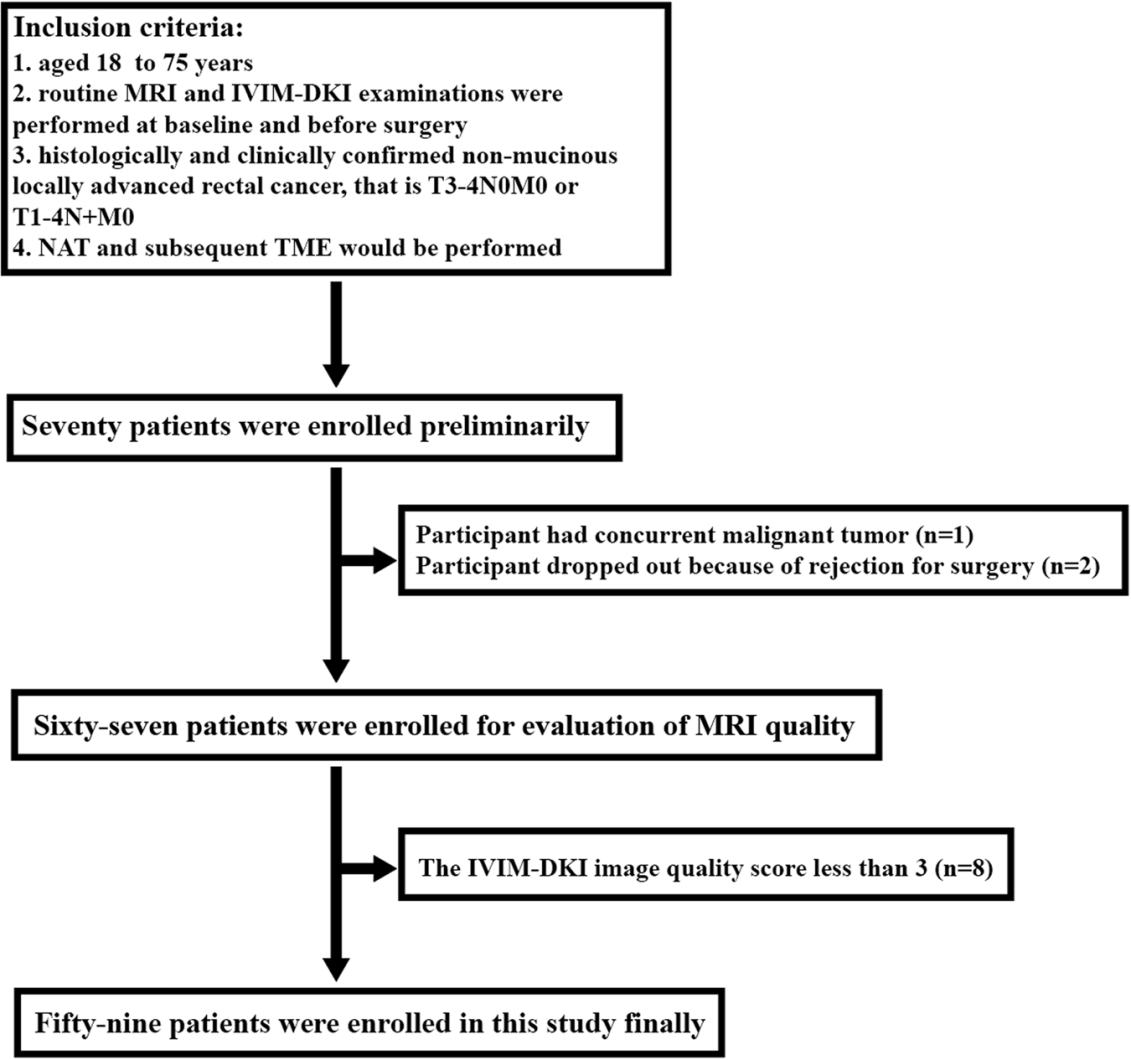
Flowchart of participant selection, inclusion, and exclusion

**Fig. 2:**
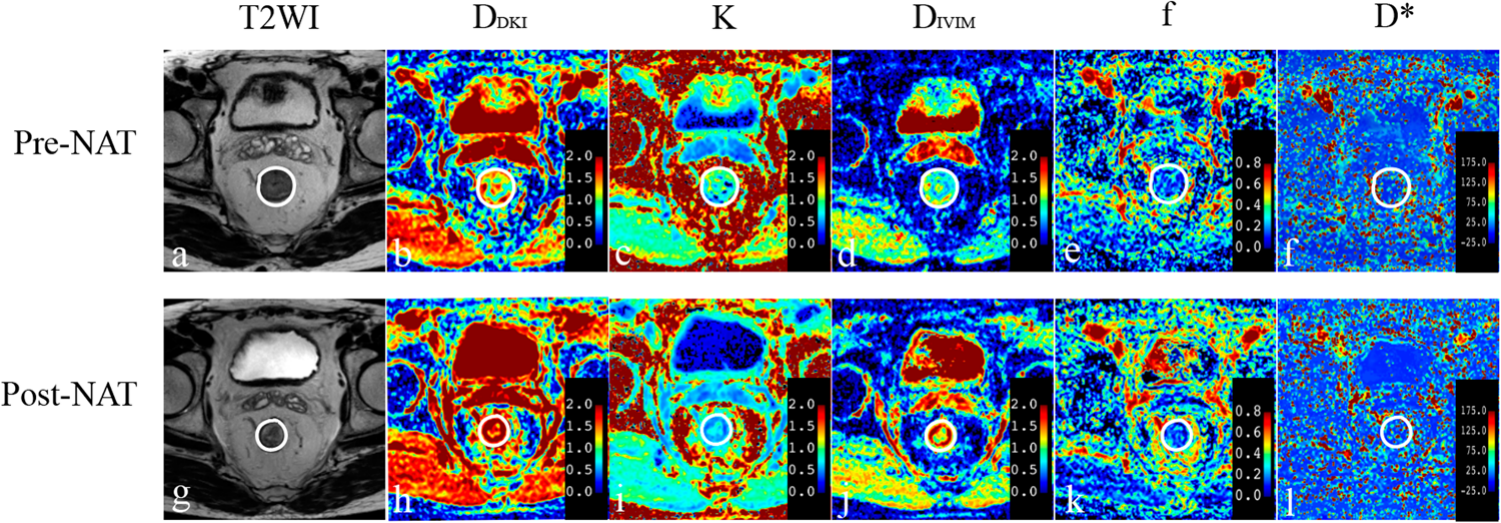
A 58-year-old man with pCR to NAT. Images in sequence are pre- and post-NAT MR imaging and IVIM–DKI parametric maps. Pre-NAT T2WI (**a**) and post-NAT T2WI (**g**) show an intermediate signal intensity rectal cancer at its largest dimension. The corresponding pre-NAT *D*_DKI_ map (**b**), pre-NAT *K* map (**c**), pre-NAT *D*_IVIM_ map (**d**), pre-NAT *f* map (**e**), pre-NAT D^*^ map (**f**), post-NAT *D*_DKI_ map (**h**), post-NAT *K* map (**i**), post-NAT *D*_IVIM_ map (**j**), post-NAT *f* map (**k**), and post-NAT D^*^ map (**l**) are shown. T2WI, T2-weighted imaging; *D*_DKI_, diffusivity of DKI; *K*, kurtosis of DKI; *D*_IVIM_, true diffusion coefficient; *f*, perfusion fraction; D^*^, pseudo-diffusion coefficient

**Fig. 3:**
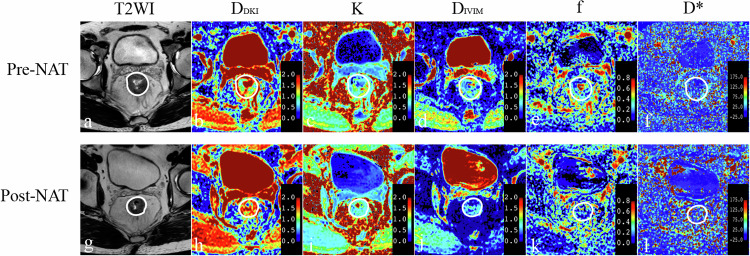
A 34-year-old man without pCR to NAT. Images in sequence are pre- and post-NAT MR imaging and IVIM–DKI parametric maps. Pre-NAT T2WI (**a**) and post-NAT T2WI (**g**) show an intermediate signal intensity rectal cancer at its largest dimension. The corresponding pre-NAT *D*_DKI_ map (**b**), pre-NAT *K* map (**c**), pre-NAT *D*_IVIM_ map (**d**), pre-NAT *f* map (**e**), pre-NAT D^*^ map (**f**), post-NAT *D*_DKI_ map (**h**), post-NAT *K* map (**i**), post-NAT *D*_IVIM_ map (**j**), post-NAT *f* map (**k**), and post-NAT D^*^ map (**l**) are shown. T2WI, T2-weighted imaging; *D*_DKI_, diffusivity of DKI; *K*, kurtosis of DKI; *D*_IVIM_, true diffusion coefficient; *f*, perfusion fraction; D^*^, pseudo-diffusion coefficient

The study flowchart is depicted in Fig. 1. A total of 59 patients (21 females and 38 males) with a median age of 58.00 years (interquartile range [IQR]: 52.00–62.00) were included in the final analysis. Demographic and clinical characteristics of the study population are summarized in Table 2. Among them, 21 patients exhibited pCR (Fig. 2) for primary tumors, while 38 patients had tumors without pCR (Fig. 3). No significant differences were observed between the pCR and non-pCR groups in terms of age, sex, tumor location, pre-NAT T stage, and pre-NAT N stage.

**Table 2:** The demographic and clinical characteristics

		Pathologic treatment response		
Variable	Total	pCR	Non-pCR	*p* value
No. of patients	59	21	38	
Age (years)	58.00 (52.00, 62.00)	55.00 (47.50, 61.50)	59.00 (53.75, 62.00)	0.173
Sex				0.788
Male	38	14	24	
Female	21	7	14	
Tumor location^*^	6.10 (4.00, 7.60)	7.00 (4.30, 7.60)	6.00 (4.00, 7.63)	0.716
Pre-NAT T stage				0.243
T3	47	15	32	
T4	12	6	6	
Pre-NAT N stage				0.305
N0	19	5	14	
N1–2	40	16	24	

* Distance of inferior border of tumor to anal verge

### Image quality assessment

Among the 70 participants meeting the inclusion criteria, one had a concurrent malignant tumor, and two withdrew from surgery. Of the remaining 67 participants who underwent image quality assessment encompassing T2WI, DWI, and IVIM–DKI imaging, 8 were excluded due to poor quality IVIM–DKI images. Interobserver agreement between the two experienced radiologists was excellent or good for T2WI (κ = 0.93, *p* < 0.05), DWI (κ = 0.89, *p* < 0.05), and IVIM–DKI (κ = 0.83, *p* < 0.05) regarding image quality scores. Figures E1 and E2 display the image quality scores of DWI and IVIM–DKI.

### Interobserver agreement

The interobserver agreement for conventional MRI assessment of CR based on T2WI and DWI was good (κ = 0.66). All measured parameters exhibited excellent or good interobserver agreement, with ICCs ranging from 0.786 to 0.960.

### Diagnostic performance of conventional MRI based on T2WI and DWI

**Fig. 4:**
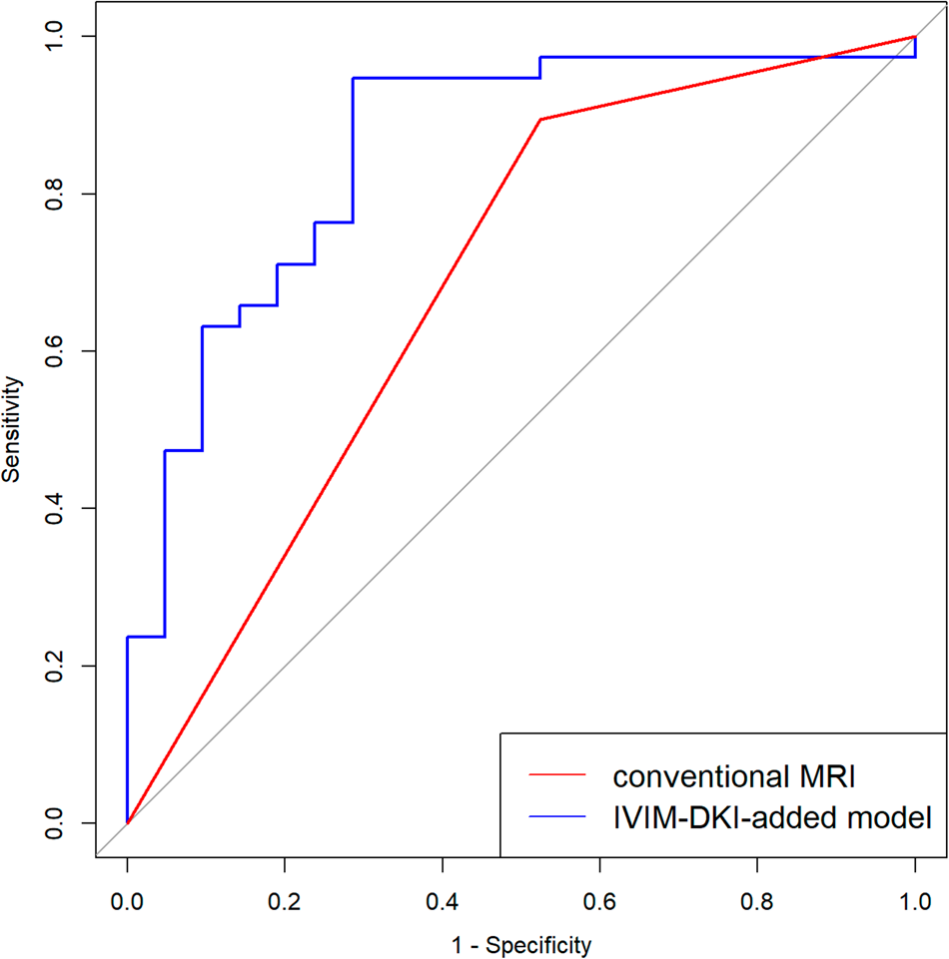
ROC curves for evaluating CR to NAT in LARC. The AUC of the IVIM–DKI-added model (combination of IVIM and DKI histogram parameters and conventional MRI) and conventional MRI (based on T2-weighted imaging and DWI) were 0.855 (95% CI: 0.749–0.960) and 0.685 (95% CI: 0.565–0.806), respectively. *p* value for the comparison of the two AUCs, *p* < 0.001

The accuracy, sensitivity, and specificity of conventional MRI based on T2WI and DWI for evaluating the CR of primary tumors were 0.746 (95% CI: 0.739–0.752), 0.895 (95% CI: 0.797–0.992), and 0.476 (95% CI: 0.263–0.690), respectively (Table 3), with an AUC of 0.685 (95% CI: 0.565–0.806) (Fig. 4).

**Table 3:** Diagnostic performance for the evaluation of CR to NAT

Method/model	Accuracy	Sensitivity	Specificity	AUC
Conventional MRI^#^	0.746 (0.739–0.752)	0.895 (0.797–0.992)	0.476 (0.263–0.690)	0.685 (0.565–0.806)
IVIM–DKI-added model^*^	0.864 (0.861–0.868)	0.947 (0.876–1.000)	0.714 (0.521–0.908)	0.855 (0.749–0.960)

Data in parentheses are 95% CIs

^#^ The conventional MRI was based on T2WI and DWI

^*^ Combination of IVIM and DKI histogram parameters (post-*K*_Skewness_ and Δ%*D*_DKI-root mean squared_), T2WI, and DWI

### Diagnostic performance of IVIM–DKI-added model

A total of 27 histogram parameters differed between the two groups. Most post-*D* and Δ%*D* histogram parameters of DKI were significantly higher in pCR patients, while most post-*K* histogram parameters were significantly higher in non-pCR patients. Detailed data of the 27 histogram parameters and their ROC curve results are listed in Table E1. Based on the two main criteria mentioned above, variables with lower *p*-values (*p* < 0.05) and higher AUCs were preferentially selected. We excluded extreme values of histogram parameters, such as minimum and maximum values, due to their susceptibility to measuring errors. Three variables—post-*K*_Skewness_, Δ%*D*_DKI-mean_, and Δ%*D*_DKI-root mean squared_—were selected for further analysis. Based on univariate logistic regression analysis, the AUCs of post-*K*_Skewness_, Δ%*D*_DKI-mean_, and Δ%*D*_DKI-root mean squared_ were 0.751 (95% CI: 0.619–0.883), 0.702 (95% CI: 0.568–0.836), and 0.682 (95% CI: 0.543–0.820), respectively.

In multivariate logistic regression analysis, post-*K*_Skewness_ and Δ%*D*_DKI-root mean squared_ were finally included in the IVIM–DKI-added model, while Δ%*D*_DKI-mean_ was excluded due to its insignificant contribution to the model. The accuracy, sensitivity, and specificity of the IVIM–DKI-added model for evaluating the CR of primary tumors were 0.864 (95% CI: 0.861–0.868), 0.947 (95% CI: 0.876–1.000), and 0.714 (95% CI: 0.521–0.908), respectively (Table 3), with an AUC of 0.855 (95% CI: 0.749–0.960) (Fig. 4).

## Discussion

In this study, the addition of IVIM–DKI histogram parameters (specifically, post-*K*_Skewness_ and Δ%*D*_DKI-root mean squared_) extracted from a unified “one-stop” IVIM–DKI scanning protocol to conventional MRI based on T2WI and DWI significantly enhanced the diagnostic accuracy for evaluating CR to NAT in LARC, with the AUC increasing from 0.685 (95% CI: 0.565–0.806) to 0.855 (95% CI: 0.749–0.960).

From a theoretical standpoint, owing to the intricate nature of tissue microstructure, water proton mobility within biological tissues exhibits non-Gaussian diffusivity [29]. Conventional DWI relies on the mono-exponential model, which fails to accurately portray the diffusion of water molecules. Conversely, advanced diffusion models like IVIM and DKI are founded on the principle of non-Gaussian motion, allowing them to faithfully capture the non-Gaussian diffusion characteristics of water molecules in biological and tumor tissues. Following NAT, rectal tumors undergo a range of radiation-induced tissue alterations including edema, inflammation, necrosis, and fibrosis, rendering the cellular microstructure more heterogeneous and complex. However, despite undergoing prolonged NAT, approximately 30% of patients may still exhibit resistance to treatment [30]. Variations in resistance levels may consequently impact treatment efficacy.

Our study revealed that most post-*D* and Δ%*D* histogram parameters of DKI were notably higher in patients exhibiting pCR compared to those without pCR, while most post-*K* histogram parameters were significantly higher in non-pCR patients. This observation may be attributed to the effective NAT-inducing changes in tumor microstructure such as necrosis, reduced cellular density, and increased extracellular space, resulting in a decrease in the degree of restricted diffusion. Consequently, we speculate that higher post-*D* and Δ%*D* values in DKI, along with lower post-*K* values, in patients with LARC after NAT correspond to lesions with a reduced likelihood of resistance to NAT. Our findings are consistent with prior research. For instance, Li et al [31] found that Δ%*D*_DKI-mean_ was informative in assessing tumoral resistance to NAT in LARC patients, while Cui et al [32] demonstrated an association between histogram parameters of *K* and important prognostic factors of rectal cancer.

Furthermore, our model included post-*K*_Skewness_ and Δ%*D*_DKI-root mean squared_, indicating that skewness and root mean squared of the histogram may reflect residual tumor information. This aligns with the findings of Babatürk et al [33], who reported a correlation between histogram skewness and the percentage of residual tumor. Similarly, Yu et al [34] discovered that DKI, coupled with entire-tumor histogram analysis, was feasible and reliable for assessing good responders to NAT in LARC. Notably, they found that the histogram parameter pre-D _DKI-10th percentile_ yielded AUC values of 0.753, with a sensitivity of 66.67% and a specificity of 77.78%.

According to a previous study [35], the DKI model appears to be the favored model in terms of model fit and repeatability. DKI demonstrates greater robustness compared to non-Gaussian diffusion models such as stretched exponential and IVIM models, as it employs polynomials to fit two unknown parameters (*D*_DKI_ and *K*) [36]. These factors may account for the superior performance of DKI parameters in our study, leading to their inclusion in our final IVIM–DKI-added model rather than IVIM parameters, despite significant differences observed in some IVIM histogram parameters between pCR and non-pCR patients. In a similar study, Yang et al [37] found that IVIM and DKI are effective in the evaluation of CR after NAT in LARC, and the parameters of DKI performed better than those of IVIM, which was consistent with our study. They acquired IVIM and DKI images separately, whereas we adopted the combined IVIM–DKI scanning protocol. Notably, pre-NAT histogram parameters were not incorporated into our model. We speculate that this is because post-NAT histogram parameters and the change ratio of parameters before and after NAT are more pertinent to postoperative pathology and prognosis in LARC. Clinical practitioners typically assess treatment efficacy by comparing parameters before and after treatment and evaluating the change ratio to adjust treatment plans.

In our study, we employed histogram analysis for both pre- and post-NAT. This approach enables the quantification of changes in tumor heterogeneity using descriptive parameters obtained from the histogram of grayscale variations within ROI, thereby providing a more accurate depiction of the tumor’s internal status [38]. As a first-order texture analysis method, histogram analysis offers greater repeatability compared to higher-order texture features [39, 40]. Moreover, we overlaid the IVIM–DKI images on T2WI, as T2WI facilitates precise localization of lesion regions and boundaries. The ROI delineation method utilized in our study further enhanced the accuracy of lesion delineation. Additionally, we adopted a unified “one-stop” IVIM–DKI scanning protocol to acquire IVIM and DKI in a single scan. This scanning approach offers two primary advantages: firstly, compared to separate IVIM and DKI scanning protocols, it reduces data redundancy, shortens scan time, and mitigates motion-related artifacts; secondly, it enables utilization of more advanced hybrid IVIM–DKI models, which can capture the complexity of tumor microstructure more accurately [41, 42]. In this study, this “one-stop” acquisition protocol achieved the desired image quality in a more efficient scan time of 5 min 24 s per patient. We infer that our new findings could apply to MRI scans with 1.5-T for the evaluation of CR to NAT in LARC, which needs adjustments in scan parameters and longer scan duration, due to the lower signal-to-noise ratio (SNR) in 1.5-T than in 3.0-T.

Our study had several limitations that need to be acknowledged. Firstly, the relatively small size of our study population may introduce statistical bias, and insufficient generalization of this technique. Consequently, larger-scale validation studies are needed. Secondly, while secondary texture features have been shown to potentially offer a more comprehensive representation of tumor heterogeneity by reflecting spatial and positional relationships between pixels and voxels, their repeatability is poor. Future studies should explore the inclusion of secondary texture features in IVIM and DKI images of rectal cancer and compare their predictive performance with histogram analysis. Thirdly, the stability of IVIM and DKI parameters depends heavily on image quality factors such as position matching and SNR, which could affect parameter measurement. Fourthly, our ROI placement was limited to the level of the largest area of suspicious residual tumor or the corresponding level to the largest area of the initial tumor, potentially introducing bias. Additionally, while the “one-stop” IVIM–DKI protocol offers advantages in reducing data redundancy, excessive *b* values may prolong scan times and increase echo time due to high *b* values in DKI. Further research is needed to determine the optimal number and interval of *b* values for this approach. Moreover, in our study, IVIM and DKI data were reconstructed separately, potentially missing important information compared to an advanced hybrid IVIM–DKI model.

In conclusion, IVIM and DKI histogram analysis offer added value in assessing CR to NAT in LARC. Superimposing the advantage of noninvasiveness, IVIM, and DKI can help mitigate uncertainty in diagnosing CR to NAT using conventional MRI.

## Supplementary information

ELECTRONIC SUPPLEMENTARY MATERIAL

## Abbreviations

ADC: Apparent diffusion coefficient
AUC: Area under the receiver operating characteristic curve
CI: Confidence interval
CR: Complete response
*D*^*^: Pseudo-diffusion coefficient
*D*_DKI_: Diffusivity of DKI
*D*_IVIM_: True diffusion coefficient
DKI: Diffusion kurtosis imaging
DWI: Diffusion-weighted imaging
*f*: Perfusion fraction
ICC: Intraclass correlation coefficient
IVIM: Intravoxel incoherent motion
*K*: Kurtosis of DKI
LARC: Locally advanced rectal cancer
NAT: Neoadjuvant therapy
pCR: Pathological complete response
ROI: Region of interest
SNR: Signal-to-noise ratio
