# Supplementary material for: Added value of histogram analysis of intravoxel incoherent motion and diffusion kurtosis imaging for the evaluation of complete response to neoadjuvant therapy in locally advanced rectal cancer
Source: Eur Radiol. 2024 Sep 19;35(3):1669–78. doi: 10.1007/s00330-024-11081-z (PMC11835893; doi:10.1007/s00330-024-11081-z)
Supplement: Supplementary file 1 — ELECTRONIC SUPPLEMENTARY MATERIAL [file 330_2024_11081_MOESM1_ESM.pdf]

Histogram analysis

IVIM and DKI histogram parameters consist of 10th percentile, 90th percentile, energy, entropy, interquartile range, kurtosis, maximum, mean absolute deviation, mean, median, minimum, range, robust mean absolute deviation, root mean squared, skewness, total energy, uniformity and variance.

(https://pyradiomics.readthedocs.io/en/latest/features.html)

$$\text{energy} = \sum_{i=1}^{N_p} (\boldsymbol{X}(i) + c)^2$$

$$\text{total energy} = \mathrm{V}_{\text{voxel}} \sum_{i=1}^{N_p} (\boldsymbol{X}(i) + c)^2$$

$$\text{entropy} = -\sum_{i=1}^{N_g} p(i) \log_2(p(i) + \epsilon)$$

Here, ϵ is an arbitrarily small positive number (≈2.2×10−16)

$$\text{mean} = \frac{1}{N_p} \sum_{i=1}^{N_p} \boldsymbol{X}(i)$$

$$\text{interquartile range} = \mathbf{P}_{75} - \mathbf{P}_{25}$$

Here $\mathbf{P}_{25}$ and $\mathbf{P}_{75}$ are the 25th and 75th percentile of the image array, respectively

$$\text{range} = \max(\mathbf{X}) - \min(\mathbf{X})$$

$$MAD = \frac{1}{N_p} \sum_{i=1}^{N_p} |\boldsymbol{X}(i) - \bar{X}|$$

$$\text{rMAD} = \frac{1}{N_{10-90}} \sum_{i=1}^{N_{10-90}} |X_{10-90}(i) - \bar{X}_{10-90}|$$

$$\text{RMS} = \sqrt{\frac{1}{N_p} \sum_{i=1}^{N_p} (\boldsymbol{X}(i) + c)^2}$$

Here, c is optional value, defined by voxelArrayShift, which shifts the intensities to prevent negative

values in X. This ensures that voxels with the lowest gray values contribute the least to RMS, instead of voxels with gray level intensity closest to 0.

$$\text{skewness} = \frac{\mu_3}{\sigma^3} = \frac{\frac{1}{N_p}\sum_{i=1}^{N_p}(\boldsymbol{X}(i) - \bar{X})^3}{\left(\sqrt{\frac{1}{N_p}\sum_{i=1}^{N_p}(\boldsymbol{X}(i) - \bar{X})^2}\right)^3}$$

Where μ_3 is the 3rd central moment

$$\text{kurtosis} = \frac{\mu_4}{\sigma^4} = \frac{\frac{1}{N_p}\sum_{i=1}^{N_p}(\boldsymbol{X}(i) - \bar{X})^4}{\left(\frac{1}{N_p}\sum_{i=1}^{N_p}(\boldsymbol{X}(i) - \bar{X})^2\right)^2}$$

Where μ_4 is the 4th central moment

$$\text{variance} = \frac{1}{N_p}\sum_{i=1}^{N_p}(\boldsymbol{X}(i) - \bar{X})^2$$

$$\text{uniformity} = \sum_{i=1}^{N_p}\boldsymbol{p}(i)^2$$

Table E1 Differences and diagnostic performance of IVIM and DKI histogram parameters between pCR and Non-pCR patients

Parameter	pCR	Non-pCR	*P* value	AUC (95% CI)	Sen (%)	Spe (%)	Acc (%)
Post-$D_{DKI\text{-}10th\ Percentile}$ ($\times 10^{-3}$ mm^2/s)	1.32 (1.08, 1.52)	1.04 (0.87, 1.32)	0.018	0.687 (0.553−0.801)	52.6	81.0	62.7
△%$D_{DKI\text{-}10th\ Percentile}$	74.21 (38.92, 115.17)	36.02(6.03,75.97)	0.026	0.677 (0.535−0.818)	44.7	90.5	61.0
Post-$D_{DKI\text{-}Interquartile\ Range}$ ($\times 10^{-3}$ mm^2/s)	0.42 (0.32, 0.57)	0.57 (0.44, 0.66)	0.043	0.660 (0.511−0.809)	78.9	52.4	69.5
Post-$D_{DKI\text{-}Mean}$ ($\times 10^{-3}$ mm^2/s)	1.73 (1.63, 1.85)	1.63 (1.46, 1.82)	0.035	0.667 (0.529−0.804)	47.4	90.5	62.7
△%$D_{DKI\text{-}Mean}$	45.58 (34.89, 67.19)	27.62 (15.60, 50.00)	0.011	0.702 (0.568 −0.836)	52.6	85.7	64.4
△%$D_{DKI\text{-}Median}$	54.03 (45.63, 69.17)	32.60 (20.26, 51.72)	0.005	0.723 (0.587−0.859)	71.1	81.0	74.6
Post-$D_{DKI\text{-}Minimum}$ ($\times 10^{-3}$ mm^2/s)	0.96 (0.36, 1.23)	0.35 (0.01, 0.91)	0.015	0.692 (0.546−0.839)	44.7	90.5	61.0
Post-$D_{Robust\ Mean\ Absolute\ Deviation}$($\times 10^{-3}$ mm^2/s)	0.19 (0.15, 0.23)	0.24 (0.19, 0.29)	0.037	0.665 (0.517−0.813)	57.9	76.2	64.4
Post-$D_{Root\ Mean\ Squared}$ ($\times 10^{-3}$ mm^2/s)	1.77 (1.71, 1.91)	1.69 (1.53, 1.88)	0.046	0.658 (0.518−0.798)	57.9	81.0	66.1
△%$D_{Root\ Mean\ Squared}$	39.18 (31.27, 68.60)	28.70 (13.13, 46.24)	0.022	0.682 (0.543−0.820)	50.0	85.7	62.7
△%$K_{90th\ percentile}$	-30.28 (-40.96, -21.40)	-19.23 (-30.93, -4.45)	0.014	0.695 (0.555−0.836)	52.6	81.0	62.7
Post-K_{Energy}	49.61 (26.11, 79.90)	103.30 (49.56, 379.20)	0.004	0.731 (0.603−0.858)	55.3	85.7	66.1
△%K_{Energy}	-80.96 (-91.91, -57.15)	-60.21 (-76.56，28.11)	0.003	0.737 (0.606−0.868)	81.6	57.1	72.9
Post-$K_{Kurtosis}$	3.73 (3.38, 7.26)	8.48 (3.70, 40.38)	0.027	0.675(0.537−0.814)	52.6	85.7	64.4
△%$K_{Kurtosis}$	12.96 (-26.53, 43.46)	81.62 (-17.01, 439.50)	0.040	0.663(0.525−0.801)	55.3	81.0	64.4
Post-$K_{Maximum}$	1.00 (0.88, 1.44)	1.64 (1.32, 11.89)	<0.001	0.793(0.670−0.916)	76.3	76.2	76.3

△%K $_{Maximum}$	-38.88 (-53.74, -16.21)	-5.45 (-42.87, 470.01)	0.013	0.697(0.561−0.832)	60.5	76.2	66.1
Post-K $_{Mean Absolute Deviation}$	0.12 (0.09, 0.19)	0.20 (0.12, 0.42)	0.003	0.733(0.603−0.863)	50.0	90.5	64.4
△%K $_{Mean Absolute Deviation}$	-46.10 (-65.63, -25.60)	-32.12 (-52.28, 103.63)	0.028	0.674(0.535−0.813)	31.6	100.0	55.9
Post-K $_{Range}$	0.77 (0.62, 1.44)	1.55 (1.12, 11.89)	0.001	0.773(0.647−0.899)	73.7	71.4	72.9
△%K $_{Range}$	-54.16 (-72.38, -10.04)	-10.20 (-48.05, 469.11)	0.012	0.699(0.564−0.835)	60.5	76.2	66.1
Post-K $_{Robust Mean Absolute Deviation}$	0.08 (0.06, 0.11)	0.10 (0.08, 0.18)	0.031	0.670(0.528−0.813)	44.7	85.7	59.3
Post-K $_{Skewness}$	-0.50 (-0.87, 0.63)	1.20 (-0.19, 5.48)	0.002	0.751(0.619 −0.883)	89.5	52.4	76.3
Post-K $_{Total Energy}$	408.80 (215.14, 658.38)	851.14 (432.18, 3124.50)	0.003	0.734(0.607 −0.861)	55.3	85.7	66.1
Post-K $_{Variance}$	0.02 (0.01, 0.07)	0.08 (0.03, 1.10)	0.001	0.752(0.624 −0.879)	84.2	57.1	74.6
△%K $_{Variance}$	-77.11 (-91.62, -29.90)	-54.10 (-76.63, 1002.97)	0.011	0.702(0.566 −0.837)	36.8	100.0	59.3
△%D $_{IVIM-Median}$	59.41 (43.71, 87.36)	37.92 (18.18, 53.46)	0.015	0.693(0.544 −0.842)	86.8	52.4	74.6

Note: 95% confidence intervals in bracket

Table E2. Scoring Criteria of Image Quality Evaluation for T2-weighted Imaging

Image Score	Scoring Criteria
5	excellent
4	good with no limitation to diagnostic quality
3	fair with no severe limitation to diagnostic quality
2	poor, albeit still interpretable
1	unacceptable with nondiagnostic images

Table E3. Scoring Criteria of Image Quality Evaluation for Diffusion-weighted Imaging

Image Score	Distortion Degree	Visibility	Motion Artifact
4	No distortion	Excellent visibility	No artifact
3	Little distortion	Visible	Slight artifact
2	Moderated distortion	Partial visibility	Moderate artifact
1	Marked distortion	Not visible	Marked artifact

Table E4. Scoring Criteria of Image Quality Evaluation for IVIM-DKI

Image Score	Scoring Criteria
5	Tumor detectable and lesion contour clearly delineated without T2- weighted image
4	Tumor detectable without T2-weighted image but contour poorly seen
3	Tumor undetectable without reference to T2-weighted image
2	Poor IVIM-DKI images quality with obvious artifacts
1	No lesions identified on T2-weighted or IVIM-DKI images

Note: IVIM-DKI = intravoxel incoherent motion and diffusion kurtosis imaging

Figure E1

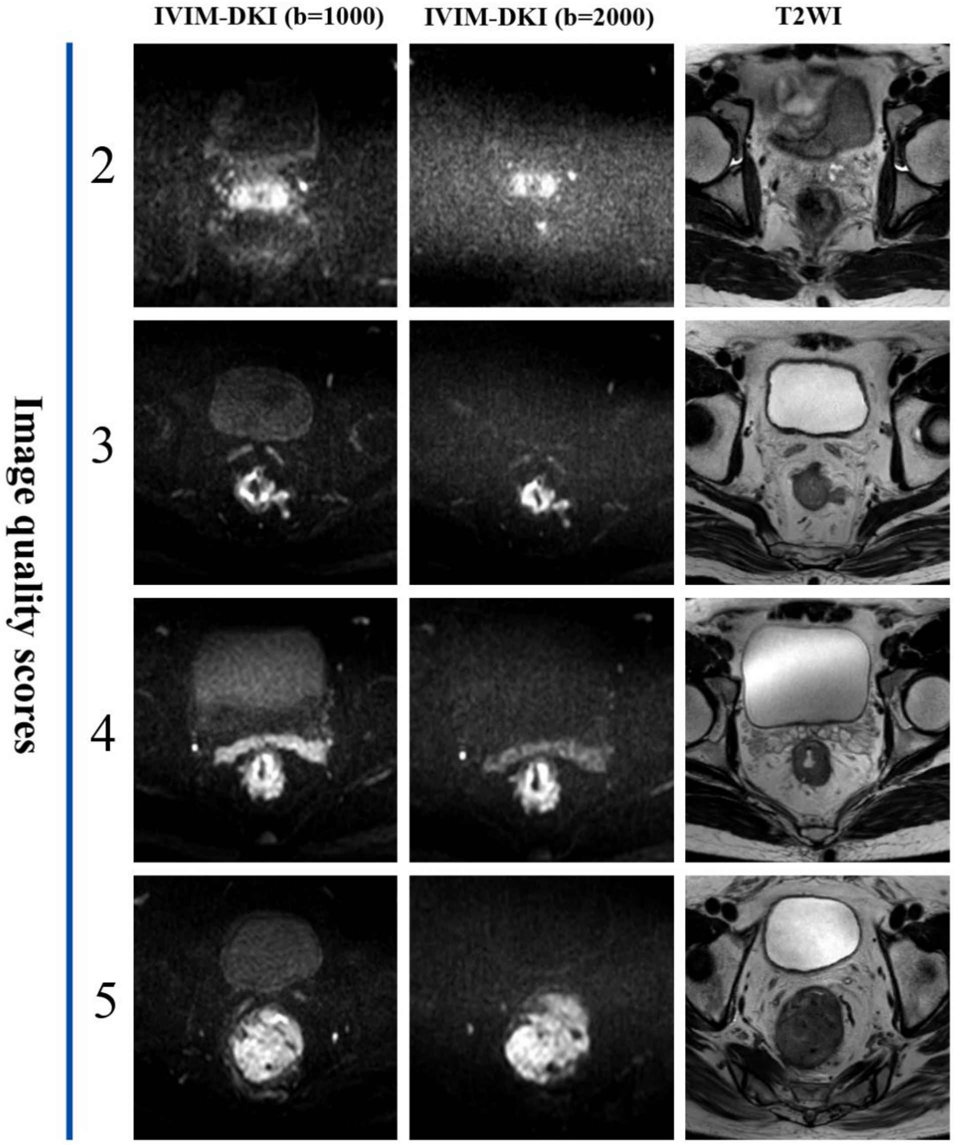

Figure E1 shows four participants with a respective IVIM-DKI image quality score of 2-5. IVIM-DKI = intravoxel incoherent motion and diffusion kurtosis imaging, T2WI = T2-weighted MRI.

Figure E2

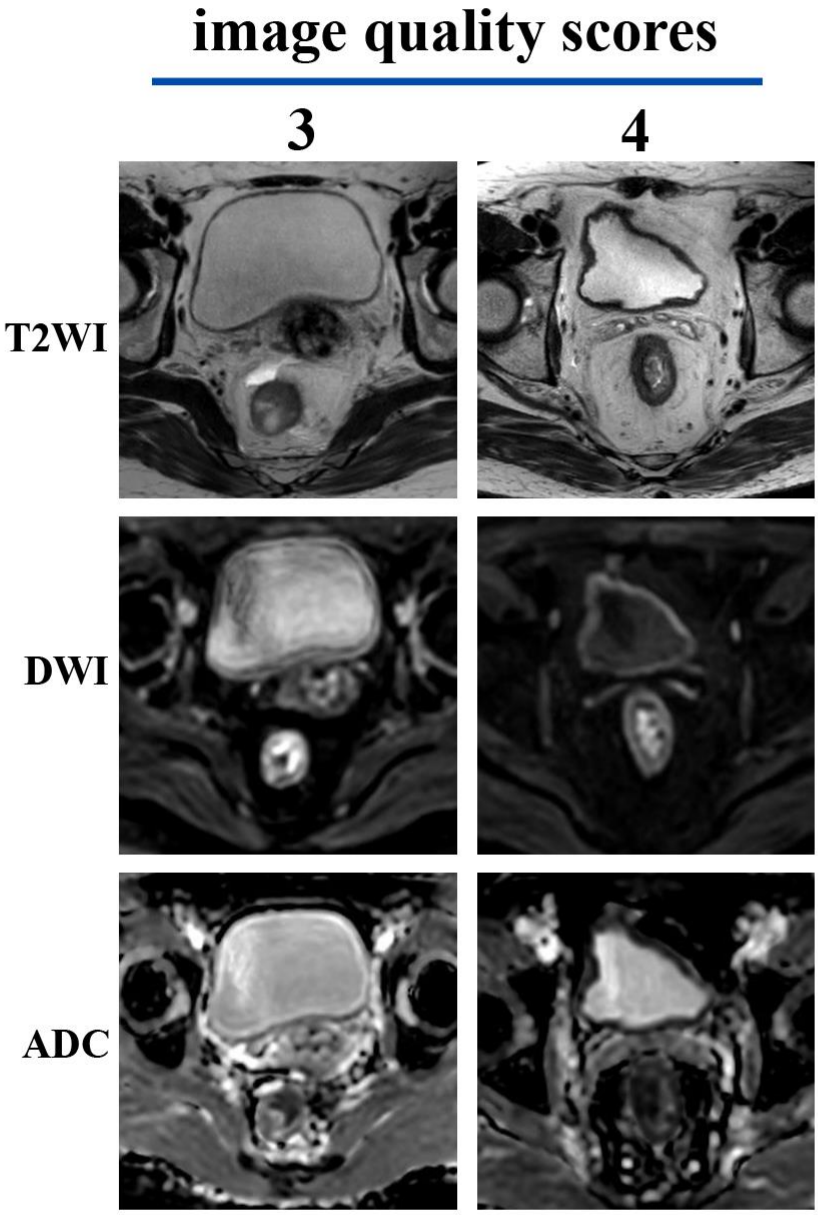

Figure E2 shows two participants with DWI image quality scores of 3 and 4.

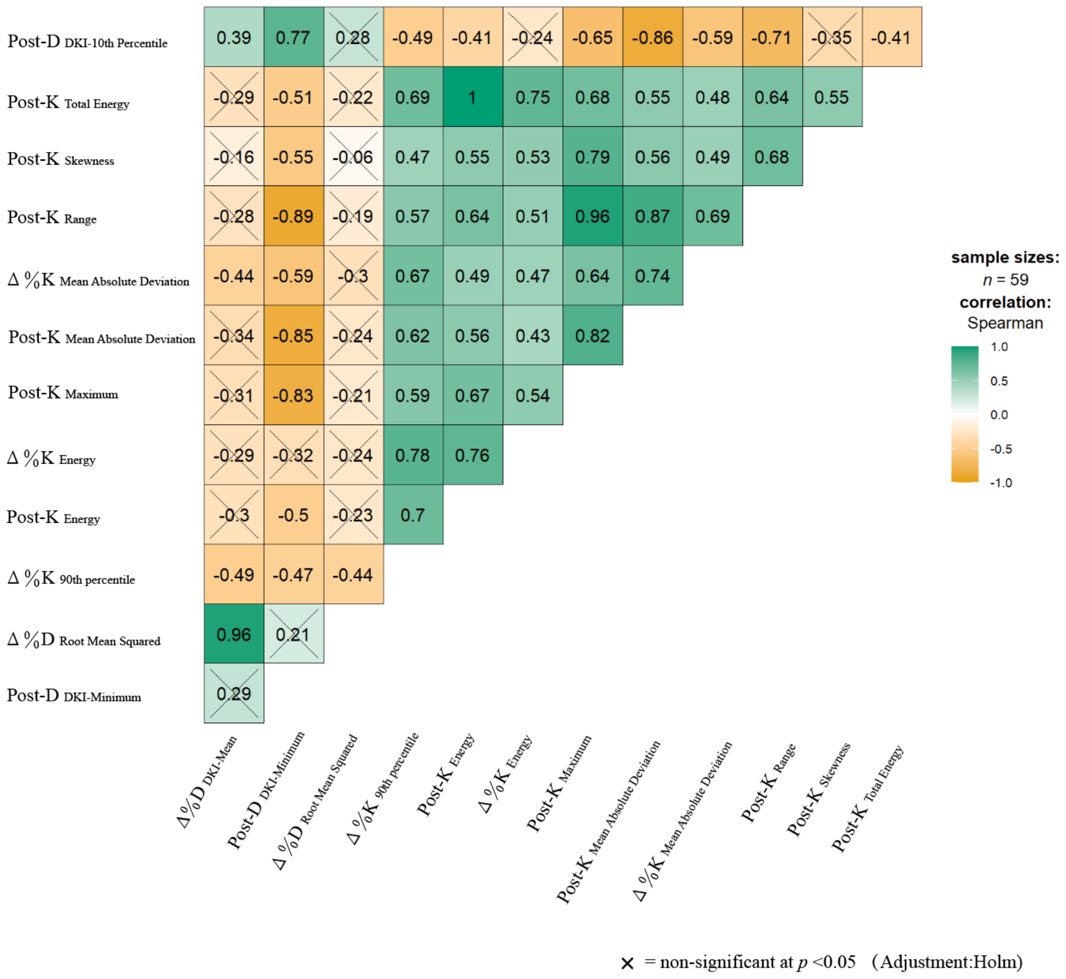

Figure E3 shows feature selection of histogram parameters.
